# Global Transcriptome Changes That Accompany Alterations in Serotonin Levels in *Caenorhabditis elegans*

**DOI:** 10.1534/g3.120.401088

**Published:** 2020-01-29

**Authors:** Johnny Cruz-Corchado, Felicia K. Ooi, Srijit Das, Veena Prahlad

**Affiliations:** Department of Biology, Aging Mind and Brain Initiative, Iowa Neuroscience Institute, 143 Biology Building, Iowa City, IA 52242-1324

**Keywords:** serotonin, transcriptome, *C. elegans*, stress, development

## Abstract

Serotonin (5-hydroxytryptamine, 5-HT), is a phylogenetically ancient molecule best characterized as a neurotransmitter that modulates multiple aspects of mood and social cognition. The roles that 5-HT plays in normal and abnormal behavior are not fully understood but have been posited to be due to its common function as a ‘defense signal’. However, 5-HT levels also systemically impact cell physiology, modulating cell division, migration, apoptosis, mitochondrial biogenesis, cellular metabolism and differentiation. Whether these diverse cellular effects of 5-HT also share a common basis is unclear. *C. elegans* provides an ideal system to interrogate the systemic effects of 5-HT, since lacking a blood-brain barrier, 5-HT synthesized and released by neurons permeates the organism to modulate neuronal as well as non-neuronal cells throughout the body. Here we used RNA-Seq to characterize the systemic changes in gene expression that occur in *C. elegans* upon altering 5-HT levels, and compared the transcriptomes to published datasets. We find that an acute increase in 5-HT is accompanied by a global decrease in gene expression levels, upregulation of genes involved in stress pathways, changes that significantly correlate with the published transcriptomes of animals that have activated defense and immune responses, and an increase in levels of phosphorylated eukaryotic initiation factor, eIF2α. In 5-HT deficient animals lacking tryptophan hydroxylase (*tph-1* *(mg280) II*) there is a net increase in gene expression, with an overrepresentation of genes related to development and chromatin. Surprisingly, the transcriptomes of animals with acute increases in 5-HT levels, and 5-HT deficiency do not overlap with transcriptomes of mutants with whom they share striking physiological resemblance. These studies are the first to catalog systemic transcriptome changes that occur upon alterations in 5-HT levels. They further show that in *C. elegans* changes in gene expression upon altering 5-HT levels, and changes in physiology, are not directly correlated.

Serotonin (5-hydroxytryptamine, 5-HT) is best characterized as a neurotransmitter in its roles modulating cognition, perception, the sleep-wake cycle, mood and emotional responses (Bauer 2015; Chaouloff 2002; Deakin 2013; Meneses 2013). A hypothesis that integrates the diverse behavioral roles of mammalian 5-HT postulates that 5-HT release serves as a ‘defense signal’ to mediate distinct adaptive responses to different types of aversive stimuli through different 5-HT pathways (Deakin and Graeff 1991; Chaouloff *et al.* 1999). However, in many organisms including mammals, 5-HT is not only present in the brain but is also abundant in peripheral tissues where its activity is not clearly connected with defense (Berger *et al.* 2009; Curran and Chalasani 2012; Azmitia 2001). For instance, in mammals although brain 5-HT does not cross the blood brain barrier, 5-HT synthesized by the intestinal enterochromaffin cells and the pineal gland enters the blood and acts on the lung, kidney, platelets, and the gastrointestinal tract to modulate cell division, cell migration, cell differentiation, glucose homeostasis, lipid metabolism, cellular respiration and other basic cell biological processes (Berger *et al.* 2009; Azmitia 2001). Whether this extended repertoire of cellular responses elicited by 5-HT share conceptual commonalities, and if so, what these may be, remain to be understood.

*C. elegans* is an ideal model system to investigate the cellular responses mediated by 5-HT (Chase and Koelle 2007; Curran and Chalasani 2012). In *C. elegans* the only site of 5-HT synthesis are neuronal cells. 5-HT synthesized by neurons is released extrasynaptically to bind 5-HT receptors in other neurons and permeates the organism through the coelomic fluid to impinge on serotonergic receptors expressed by non-neuronal tissues throughout the body. Thus, in this invertebrate, modulating 5-HT levels in neurons changes 5-HT-induced responses in neuronal circuits as well as in peripheral tissue (Chase and Koelle 2007; Curran and Chalasani 2012; Sze *et al.* 2000). A deletion in *tph-1*, the rate limiting enzyme for 5-HT synthesis in neurons, and the sole tryptophan hydroxylase gene, abolishes all 5-HT in *C. elegans* and has allowed the examination of the systemic effects of 5-HT deficiency (Sze *et al.* 2000). Alternatively, exposing *C. elegans* to exogenous 5-HT causes its uptake into 5-HT producing neurons allowing the study of physiological and behavioral effects of excess 5-HT. These tools have facilitated numerous insights into *C. elegans* 5-HT biology (Jafari *et al.* 2011).

In *C. elegans*, 5-HT modifies the sensory and motor output of neurons in varying circuitry to regulate chemotaxis, thermotaxis, motility rates in response to stimuli, egg-laying rates, food search behavior, mating behavior and learning and experience-dependent behavioral plasticity (Hobert 2003; Hukema *et al.* 2008; Matsuura *et al.* 2013; Nuttley *et al.* 2002; Rankin 2006; Saeki *et al.* 2001; Tsui and van der Kooy 2008; Bargmann 2006a; Bargmann *et al.* 1993; Colbert and Bargmann 1997; Iwanir *et al.* 2016; Lee *et al.* 2017; Loer and Kenyon 1993; Brewer *et al.* 2019; Carnell *et al.* 2005; Desai and Horvitz 1989; Hapiak *et al.* 2009; Wang *et al.* 2017; Flavell *et al.* 2013). 5-HT is required for phenotypic plasticity, and the lifespan extension seen upon modulating pathways that increase longevity such as the insulin like signaling pathway (*daf-16*), or mitochondrial function (*isp-1* etc.) require 5-HT (Cunningham *et al.* 2014; Petrascheck *et al.* 2007; Sze *et al.* 2000; Ye *et al.* 2014; Yin *et al.* 2014; Zarse and Ristow 2008). In *C. elegans* as in mammals, 5-HT is released from serotonergic neurons upon exposure to threats and the acute increase in 5-HT levels activates aversive behaviors (Ooi and Prahlad 2017; Bargmann 2006b; Zhang *et al.* 2005), and modulates key stress responsive transcriptional pathways (Ooi and Prahlad 2017; Tatum *et al.* 2015; Berendzen *et al.* 2016; Zhang *et al.* 2018). However, increasing 5-HT levels also mimics food signals, and facilitates exit from dauer, promotes feeding and food-dwelling behavior, increases protein synthesis, and modulates *C. elegans* metabolism, energy homeostasis and lipid accumulation consistent with being well-fed (Chalasani *et al.* 2007; Chao *et al.* 2004; Cunningham *et al.* 2014; Diana *et al.* 2017; Gomez-Amaro *et al.* 2015; Harris *et al.* 2011; Nuttley *et al.* 2002; Song *et al.* 2013; Sze *et al.* 2000; Lee *et al.* 2011; Palamiuc *et al.* 2017; Rivard *et al.* 2010; Srinivasan *et al.* 2008; Entchev *et al.* 2015; Jafari *et al.* 2011; Lee *et al.* 2017; Noble *et al.* 2013). Conversely, *tph-1* *(mg280)* *II* mutant animals display phenotypes resembling food-deprived wild-type animals, such as the constitutive activation of the insulin/IGF-like signaling pathway and the *C. elegans* FOXO ortholog DAF-16, reduced mating, retention of eggs, and an increase in lipid storage (Liang *et al.* 2006; Sze *et al.* 2000; Srinivasan *et al.* 2008). 5-HT deficiency as occurs in *tph-1* *(mg280) II* mutant animals also impairs the ability of the animal to modulate neuronal circuit function in response to environmental change and affects the normal behavioral, physiological and transcriptional responses to environmental stimuli. As with food-deprived animals, *tph-1* animals also appear to have longer reproductive lifespans (Petrascheck *et al.* 2007, 2009; Ye *et al.* 2014). These studies have therefore largely supported the premise that in *C. elegans*, increases in endogenous 5-HT levels mimic food signals to promote growth and differentiation, and 5-HT deficiency phenocopies food-deprivation. However, as described above, discrepancies remain (Zarse and Ristow 2008; Ooi and Prahlad 2017; Tatum *et al.* 2015; Berendzen *et al.* 2016).

Here, we took advantage of the ability to manipulate 5-HT levels in *C. elegans* and employed RNA-Seq to investigate gene expression changes that occur upon altering 5-HT levels. We then compared the transcriptome changes to published datasets from starved animals, animals harboring an *eat-2* mutation that limits food intake, animals harboring a mutation in the alpha subunit of AMP-activated kinase (*aak-2*) that physiologically resemble animals with elevated 5-HT levels, and with the >200 transcription datasets from the SPELL (Serial Pattern of Expression Levels Locator; https://spell.wormbase.org/) *C. elegans* database (Figure 1A). Our results indicate that both the deficiency of 5-HT, as well as its excess cause global changes in the transcriptome predicted to affect numerous tissue-types. While the RNA-seq datasets presented here show good agreement with published transcriptomes from animals treated with a 5-HT antagonist, mianserin (Rangaraju *et al.* 2015a; Rangaraju *et al.* 2015b), Gene Ontology analysis and comparison with the various published transcriptomes showed that gene expression changes in animals with excess 5-HT correlates best with gene expression changes from animals that have activated defense responses, and RNA-seq data from *tph-1* *(mg280) II* animals was enriched for genes related to development and chromatin function. In addition, comparisons of the transcriptomes of animals with excess 5-HT or 5-HT deficiency (*tph-1* *(mg280) II*) and published transcriptional profiles of *C. elegans* mutants which they phenotypically resemble, showed no significant overlap, suggesting that the similarity in the phenotypes of these animals may stem from commonalities other than gene expression changes.

**Figure 1:**
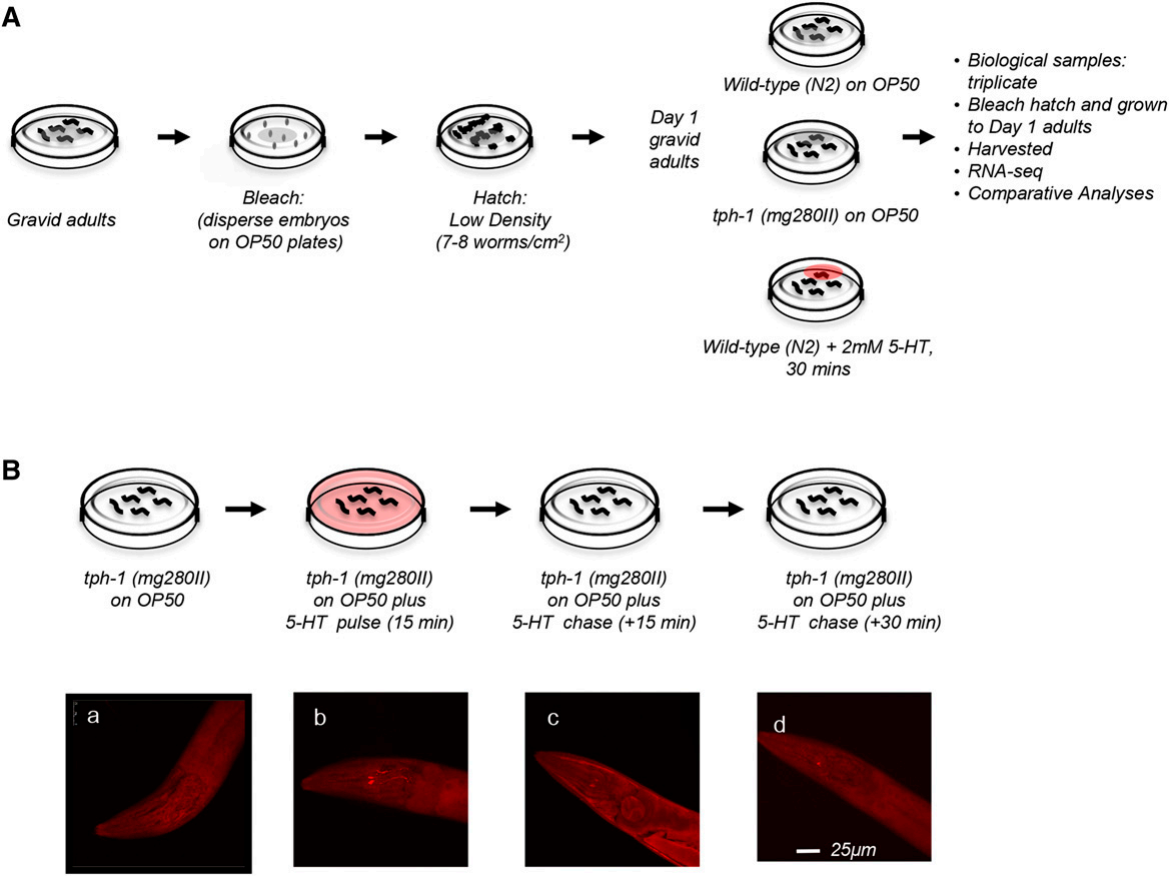
Experimental design. (A) Schematic of the samples and treatments used for RNA-seq. (B) Top: schematic of ‘pulse-chase’ experiment to determine the kinetics of exogenous 5-HT uptake and release in *C*. *elegans*. *tph-1* *(mg280) II* were exposed to a ‘pulse’ of exogenous 5-HT, by transferring them for 15 min as Day 1 adults onto OP50-seeded plates containing 2mM 5-HT. This was followed by a ‘chase’, implemented by transferring the 5-HT-exposed *tph-1* *(mg280)* *II* animals to plates seeded with OP50 without 5-HT. Bottom: Micrographs of confocal projections showing the heads of *tph-1* *(mg280)* *II* animals stained with antibodies against 5-HT. (a) control, untreated *tph-1* *(mg280)* *II*. Note the lack of staining (b) *tph-1* *(mg280)* *II* exposed to the 15 min pulse (c) *tph-1* *(mg280)* *II* exposed to 5-HT for 15 min followed by 15 min on OP50 with no 5-HT (d) *tph-1* *(mg280)* *II* exposed to 5-HT for 15 min followed by 30 min on OP50 with no 5-HT. Scale bar = 25 µm.

## Materials and Methods

### C. elegans strains

The following strains were obtained from the Caenorhabditis Genetics Center (CGC): Bristol N2 (wild type) and MT15434 *tph-1* *(mg280) II and AQ2050* *lite-1*(*ce314*); *ljIs102* [*tph-1*; *ChR2*::*YFP;unc-122*::*gfp]*. Strains were growth in standard conditions at 20 C.

### Exogenous serotonin (5-HT) treatment and validation

Exogenous serotonin treatment was modified from Jafari *et al.* (Jafari *et al.* 2011). A serotonin solution (catalog no. 85036, Sigma-Aldrich) of 2mM in sterile water was dropped onto the surface of OP50 bacterial lawns (such that the lawns were fully covered in serotonin) on NGM plates and dried for ∼2 hr at room temperature before use. Worms were transferred to the serotonin treated plates and let sit at RT for 30 min.

### RNA isolation, library preparation and sequencing

Age synchronized day 1 adult wild-type, wild type 5-HT treated and *tph-1* *(mg280) II* animals were harvested for RNA extraction. Total RNA was extracted from biological triplicates of the three treatments groups (N2, N2 +5-HT, *tph-1*). Sample lysis was performed using a Tissuelyser and a Trizol-chloroform based method was used in conjunction with the Zymo RNA Clean & Concentrator kit to obtain RNA. The Illumina TruSeq stranded mRNA kit was used to obtain stranded mRNA via Oligo-dT bead capture, and cDNA libraries were prepared from 500ng RNA per sample. Use of stranded cDNA libraries have been shown to maximize the accuracy of transcript expression estimation, and subsequent differential gene expression analysis. Each sample was multiplexed on 6 lanes of the Illumina HiSeq 4000 sequencer, generating 2x150bp paired-end reads, with about 43 to 73 million reads per sample.

### RNA-Seq analysis

FASTQC was used to evaluate the quality of the sequences. The reads were trimmed of adapter contamination and 20 bp from the 5′ and 3′ ends were removed using TrimGalore Version0.6.0 (www.bioinformatics.babraham.ac.uk/projects/trim_galore/) . Only reads with a base quality of >Q25 were maintained. Hisat2 (Pertea *et al.* 2016) was used to map the trimmed reads to the *C. elegans* genome release 35 (WBcel235). On average, 99.4% of the reads mapped to the reference genome. Assemblies of the sequences were done with Stringtie (Pertea *et al.* 2015) using the gene annotation from Ensembl WBcel235 (Zerbino *et al.* 2017). Deseq2 (Love *et al.* 2014) was used for differential expression analysis. Genes with low read counts (n < 10) in the threes set of samples (N2, N2 +5HT and *tph-1*) were removed from the analysis. A total of 17617 transcripts annotations (genes, and pseudogenes) were included for the differential expression analysis. Genes differentially expressed at an FDR < 0.01 were considered significant. To compare the global expression between treatment-groups we used the normalized counts coupled with the Log_10_ transformation and then performed two-tailed T-tests. The comparison between the significant dysregulated genes was performed in the same way as the global expression. We performed Principal component analysis (PCA) by using normalized counts coupled with the Variance Stabilization transformation (VST). The PCA was performed using the top 100 genes with the highest variance in read counts samples.

### GO analysis

The package ClusterProfiler was used to perform a Gene Ontology (GO) analysis (Yu *et al.* 2012) on the significant dysregulated genes. After applying a hypergeometric test (Yu *et al.* 2012) the GO terms with pvalue < 0.01 and Q-value < 0.05 were considered significantly enriched. GO annotations for C. elegans were obtained from R package org.Ce.eg.db: Genome wide annotation for Worm (Carlson 2018).

### Tissue expression

The tissues expression data were downloaded and collected from http://worm.princeton.edu/(Kaletsky *et al.* 2018). The clustered heatmaps were generated using the R package ‘Pheatmap’ (Kolde 2019).

### Correlation with SPELL datasets and keyword analysis

The gene expression data from the SPELL database was downloaded from https://spell.wormbase.org/ (SPELL Version 2.0.3). We filtered out the SPELL datasets that appeared to consist of absolute values, and not to have the expression data in a log fold-change scale. This was assessed by the lack of negative values in some of the SPELL datasets. However, the use of unfiltered data led to a larger set of correlated datasets, but remained restricted to the same categories, and surprisingly, did not change any conclusions of the analysis. Of the original 400 datasets, 230 SPELL datasets were obtained following filtration, and were used in the analysis. The expression data, in log fold-change scale, from significantly dysregulated genes (FDR <0.01), from our 5-HT and *tph-1* experiments was used for the comparison. Pearson’s correlations were performed between each independent experiment in the SPELL database and the *tph-1* or the *5-HT* data respectively. The p-values values were corrected using the Benjamini-Hochberg procedure (Benjamini and Hochberg 1995) and a threshold of *P* < 0.05 was established to determine the significant correlations. Scatterplots of the correlations were generated using the R package ‘ggpubr’ (Kassambara 2019). SPELL datasets containing at least one independent experiment that showed a significant positive correlation with *tph-1* or 5-HT RNA-seq data were used to perform the keyword analysis. Negative correlations were not considered. In the case of the 5-HT treatment, this generated 18 datasets while in the cases of *tph-1*, 9 datasets were generated. The keywords (22 in total) were extracted from the “description” and “topics’ section of the SPELL database through visual inspection. We then used the R package ‘base’ (R Core Team 2019) to search the total analyzed SPELL datasets (230), the 18 datasets that correlated with the gene expression from 5-HT treated animals, and the 9 datasets that correlated with gene expression from *tph-1* animals, to obtain the global (SPELL) percentages, and specific (*tph-1* and 5-HT treatment) percentages. Multiple keywords were allowed to match a dataset, but only once. We then calculated the significant enrichment (p<0.05) of each unique keyword in the treatment groups (*tph-1* and 5-HT) compared to the whole SPELL datasets by using a hypergeometric test.

### Overlap between different datasets

To analyze the overlap between the genes and their significance, we performed a hypergeometric test using the GeneOverlap package (Shen and Sinai 2019). The similarly between the groups of genes was also analyzed by calculating the Jaccard Index (Shen and Sinai 2019). Venn Diagrams were generated using the R package ‘VennDiagram’ and Dumbbell graphs were generated using the packages ‘ggalt’ and ‘bbplot’ (Chen 2018; Stylianou *et al.* 2019; Rudis *et al.* 2017). We calculated the overlap between the RNA-seq data obtained from *tph-1* animals and 5-HT treated animals with gene expression data obtained from five published datasets. The gene expression changes in *eat-2* (*ad465*) animals was obtained from Heestand *et al.* (2013). We used the published Log_2_(fold-change) gene expression change between *eat-2* and the untreated control. The gene expression changes in *aak-2* (*gt33*) animals was obtained from Shin *et al.* (2011). We used the published Log_2_(fold-change) gene expression changes between the *aak-2* (unstressed) and untreated controls. The mianserin data were obtained from Rangaraju *et al.* (2015b). From this paper we used two datasets: the first, from wild-type animals treated with mianserin (50 µM) and harvested five days after the treatment, and the second, from wild-type animals treated with mianserin (50 µM) and harvested ten days after treatment. For our analysis we used the genes determined by the authors to be changing in response to the mianserin treatment and not aging. The gene expression changes that occur upon starvation were from Harvald *et al.* (2017). We used the changes in expression between the wild-type animals starved for 16 hr and the untreated control. To transform the starvation data into a Log_2_(fold-change) scale we downloaded and reanalyzed the RNA-seq files (GEO: GSE98919), using the same pipeline described in the sub-section: **RNA-Seq analysis.**

### Western analysis for eIF2-α levels

Optogenetic experiments were performed according to previously published methods (Ooi & Prahlad 2017). Briefly, experimental plates (ATR+) were made from 100 mM ATR (product no. R2500, Sigma-Aldrich) stock dissolved in 100% ethanol and then diluted to a final concentration of 2.5 mM into OP50 culture and 200µl was seeded onto a fresh NGM plate. Control (ATR-) plates were seeded at the same time with the same culture without adding ATR. All plates were allowed to dry overnight in the dark before use. The *C. elegans* strain AQ2050 was used for this experiment. About 10 L4s were harvested on to ATR+ and ATR- plates and the experiment was carried out with day 1 adults. All plates were kept in the dark and animals were allowed to acclimatize to room temperature (20° to 22°) for about 30 min. before starting the experiment. Animals were illuminated with blue light for 30 sec at a 6.3X magnification using an MZ10 F microscope (Leica) connected to an EL6000 light source (Leica) and harvested at different time points as indicated in sterile water and snap-frozen immediately in liquid nitrogen for western blot experiment. For western blot analysis, cell lysates were prepared in 1X Laemmli sample buffer (catalog no. 1610737, Bio-Rad) supplemented with 10% β-mercaptoethanol and then incubated in boiling water for 30 min. Whole-worm lysates were resolved on 10% SDS-PAGE gels and transferred onto nitrocellulose membrane (catalog no. 1620115, Bio-Rad). Membranes were blocked with Odyssey Blocking Buffer (part no. 927-50000, LI-COR). Immunoblots were imaged using LI-COR Odyssey Infrared Imaging System (LI-COR Biotechnology, Lincoln, NE). Rabbit anti-phospho (Ser51)-eIF2-α antibody (catalog no. 9721S, Cell Signaling Technology) was used to detect the level of phosphorylated eIF2-α. Mouse anti-α-tubulin primary antibody (AA4.3), developed by C. Walsh, was obtained from the Developmental Studies Hybridoma Bank (DSHB), created by the National Institute of Child Health and Human Development (NICHD) of the National Institutes of Health (NIH), and maintained at the Department of Biology, University of Iowa. The following secondary antibodies were used: Sheep anti-mouse IgG (H&L) Antibody IRDye 800CW Conjugated (catalog no. 610-631-002, Rockland Immunochemicals) and Alexa Fluor 680 goat anti-rabbit IgG (H+L) (catalog no. A21109, Molecular Probes, Invitrogen). LI-COR Image Studio software was used to quantify protein levels in different samples, relative to α-tubulin levels. Fold change of protein levels was calculated relative to untreated controls.

### Data availability

RNA-seq reads of the *tph-1* and wild-type (N2) animals have been deposited in the NCBI Sequence Read Archive (SRA) under the SRA accession numbers SRX6955121- SRX6955138, and in the BioProject accession number PRJNA576016 in the NCBI BioProject database. RNA-seq reads of the 5-HT treated animals are deposited in the NCBI Sequence Read Archive (SRA) under the SRA accession numbers SRX7284354 - SRX7284356, and in the BioProject accession number PRJNA594152 in the NCBI BioProject database. Also see File S1 (List of genes differentially expressed at FDR <0.01; Supplemental materials). All *C. elegans* strains used in this study are available upon request. Supplemental material available at figshare: https://doi.org/10.25387/g3.11344037.

## Results

### An acute increase in serotonin levels in C. elegans results in a net decrease in gene expression levels

5-HT applied exogenously in *C. elegans* is taken up into serotonergic neurons and released into the coelomic cavity increasing 5-HT levels within the animal (Jafari *et al.* 2011). To better estimate the dose and temporal kinetics of the 5-HT release from neurons we exposed *tph-1* mutant animals that are devoid of 5-HT to exogenous 5-HT added to the bacterial lawn for 15 min, and followed the kinetics of its uptake and transit through neurons by immunofluorescence staining using anti-5-HT antibodies (Figure 1B). In control *tph-1* animals, 5-HT is not visible in any of the tissues. Upon exposure to a ‘pulse’ of 2mM 5-HT for 15 min, 5-HT was visible in the NSM neurons, neurons that typically synthesize 5-HT, and are partially exposed to the external environment (Figure 1B). Animals were then moved to plates without 5-HT to ‘chase’ the 5-HT signal and processed for immunofluorescence every 15 min to assess the loss of 5-HT. Results from this assay suggested that 2mM 5-HT was taken up and released by serotonergic neurons within 30 min and this time and concentration would be sufficient to raise levels of endogenous 5-HT. Therefore, to test the transcriptional changes elicited by acute increases in 5-HT we treated populations of Day 1 adult animals with 2mM 5-HT for 30 min, and subsequently processed them for RNA-seq (Figure 1A).

**Figure 2:**
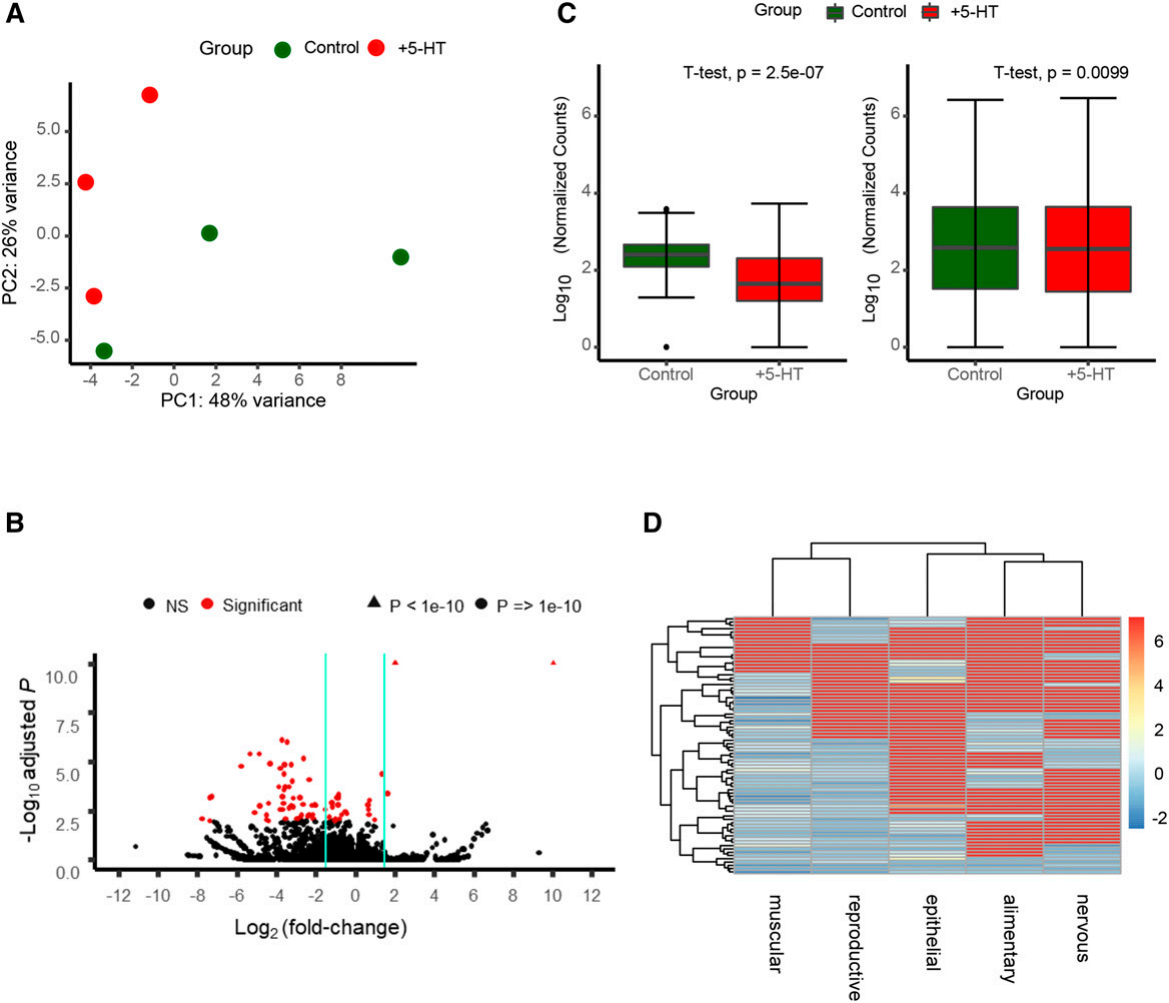
RNA-seq analysis reveals significant changes in expression in 5-HT treated animals. (A) Principal Component Analysis (PCA) plot showing the variability between the biological replicates of control untreated animals and 5-HT treated animals. (B) Volcano Plot highlighting genes with significant changes in expression (FDR-adjusted *P* < 0.01; red) or non-significant changes (black) between 5-HT-treated animals and control untreated animals. Vertical lines encompass the genes with a Log_2_(fold change) less than 1.5. In the interest of space, genes with adjusted p-value < 1e^-10^ are shown as triangles at the top of the plot. (C) Boxplots showing the differences in normalized counts per gene (log_10_ transformed) between wild type (N2) 5-HT treated animals and control untreated animals. Left Panel shows the comparison between genes that were differentially expressed (FDR-adjusted *P* < 0.01). Right Panels shows the comparison of the whole transcriptome (significant and non-significant). p-value: two tailed *t*-test. Dots = outliers. (D) Cluster Heatmap showing the expression levels in different tissues of the significantly (FDR-adjusted *P* < 0.01) dysregulated genes. Comparisons were made using data obtained from http://worm.princeton.edu. Rows and columns are clustered based on similarity in predicted expression patterns. Red-blue gradient indicates the average predicted tissue expression. red = highest predicted expression; blue = lowest predicted expression.

Animals were synchronized by bleach-hatching, and RNA was harvested from Day 1 adult animals (Figure 1A). Biological triplicates of each genotype were analyzed. The biological replicates mostly clustered by treatment as shown by Principal Component Analysis (PCA) of k-means of the samples, although the wild-type replicates did present with higher variability between samples, and the 5-HT effects were small, albeit significant (Figure 2A). Elevated 5-HT triggered the differential expression of 78 genes at a stringent FDR of 0.01 (File S1). Among these, 68 genes were downregulated and 10 upregulated (Figure 2B). Table 1 and 2 show lists of the 10 upregulated genes and the 10 most downregulated genes (Log_2_-fold changes). Remarkably, although the majority of the 10 upregulated genes were not well annotated in Wormbase (https://www.wormbase.org/#012-34-5), a manual examination of their involvement in biological pathways showed that almost all were modulated by stress. Four of the 10 genes are known targets of the stress-induced transcription factor heat shock factor 1 (HSF-1) previously shown to be activated by optogenetic release of 5-HT from neurons. These included the two *hsp70* genes *F44E5.4* and *F44E5.5* (Prahlad *et al.* 2008; Tatum *et al.* 2015; Labbadia and Morimoto 2015; Li *et al.* 2016), *Y94H6A.10* (Li *et al.* 2016), and *W02D9.10* (GuhaThakurta *et al.* 2002), genes of unknown function. Other stress-related upregulated genes were *dpf-6*, a type IV dipeptidyl peptidases that may be involved in neuropeptide processing and is a direct target of the hypoxia-inducible transcription factor HIF-1 (Shen *et al.* 2005), *Y4C6B.2*, an amino acid transporter, whose expression levels is upregulated upon oxidative stress induced by juglone (Przybysz *et al.* 2009), *C04E12.10*, an ortholog of human N-glycanase 1 (NGLY1), an enzyme linked to the degradation of dysfunctional proteins, that in *C. elegans* is upregulated by DAF-16/FOXO and SIR-2.1 upon innate immune activation (Nag *et al.* 2017), and F19B2.5, a gene of unknown function regulated by *daf-10* (Gaglia *et al.* 2012). Also upregulated was *egl-13*, the human ortholog of SOX13 (Gramstrup Petersen and Pocock 2013; Gramstrup Petersen *et al.* 2013). In C. elegans *egl-13* controls oxygen (O_2_) and carbon dioxide (CO_2_) sensing by the BAG and URX neurons which in turn, are modulated by 5-HT (Chang *et al.* 2006). Among the top downregulated genes were *cutl-9* and *cut-3*, that encode poorly characterized components of the zona pellucida (Sapio *et al.* 2005). Many of the remaining genes were not well annotated. Tissue- expression predictions extracted using information from the website, http://worm.princeton.edu (Kaletsky *et al.* 2018) showed that the genes differentially expressed upon exposure to 5-HT were predicted to be expressed in multiple tissues (Figure 2D).

**Table 1:** Upregulated Genes in 5-HT treated animals compared to untreated controls

Gene	Function	Log_2_ (fold-change)	FDR
K10D3.6	N.A.	10.05414	1.01E-12
F44E5.4	Encodes a member of the Hsp70 family of heat shock proteins	2.039188	6.6E-15
F44E5.5	Encodes a member of the Hsp70 family of heat shock proteins	2.025589	6.6E-15
Y4C6B.2	Predicted to encode a protein with the following domain: Amino acid transporter, transmembrane domain.	1.618002	0.000411
C04E12.10	N.A.	1.335383	3.98E-05
dpf-6	Predicted to have aminopeptidase activity and serine-type peptidase activity	0.948808	0.008948
Y94H6A.10	N.A.	0.705082	0.000894
egl-13	Ortholog of human SOX13 (SRY-box 13), SOX5 (SRY-box 5), and SOX6	0.663934	0.004711
W02D9.10	N.A.	0.659819	0.002569
F19B2.5	Predicted to have ATP binding activity	0.62671	0.001531

**Table 2:** Downregulated Genes in 5-HT treated animals compared to untreated controls

Gene	Function	Log_2_ (fold-change)	FDR
clec-230	Predicted to have carbohydrate binding activity	−7.78505	0.008029
ZC84.1	Predicted to have serine-type endopeptidase inhibitor activity	−7.40897	0.009795
fbxa-137	N.A.	−7.39114	0.000654
T02C12.4	N.A.	−7.30527	0.000578
F09F9.2	N.A.	−5.79695	1.61E-05
pqn-73	Predicted to contain a glutamine\/asparagine (Q\/N)-rich (’prion’) domain	−5.35092	3.91E-06
cutl-9	Predicted to encode a protein with Zona pellucida domain	−5.12811	0.003767
R09E10.5	Ortholog of human KATNAL2 (katanin catalytic subunit A1 like 2); predicted to encode a protein with the following domains: NIDO domain and AMOP domain	−4.90305	3.9E-06
Y41D4B.6	Predicted to encode a protein with the following domain: Protein Lag2	−4.87421	0.001762
cut-3	Predicted to encode a protein with Zona pellucida domain	−4.53313	0.008948

### The pattern of gene expression changes that occur upon increasing serotonin levels are most related to changes that occur during a stress response in C. elegans

**Figure 3:**
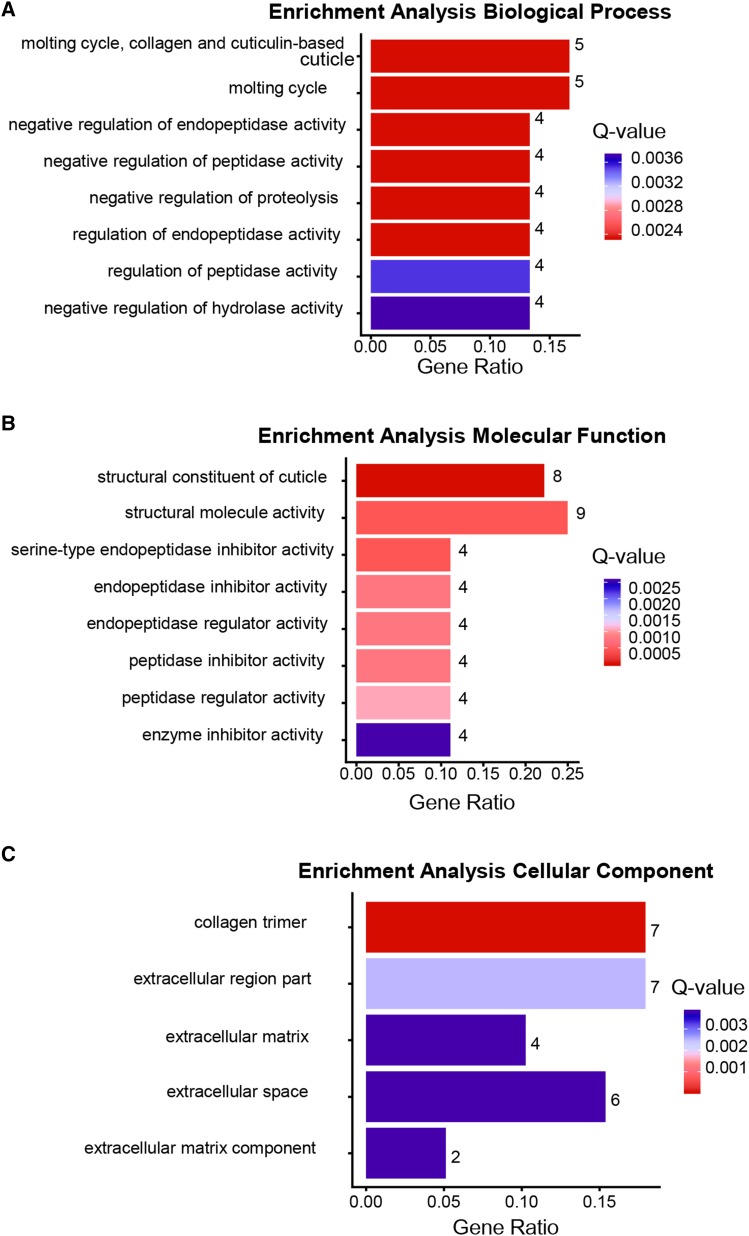
Gene Ontology of differentially expressed genes in 5-HT treated worms. Top functional categories enriched in Biological Process (A), Molecular Function (B), Cellular Component (C). x axis: gene ratios. Numbers at the end of the bar indicate the number of genes present in each category; Color bar: Q-values. Red = lowest Q-values and blue = highest Q-values.

**Figure 4:**
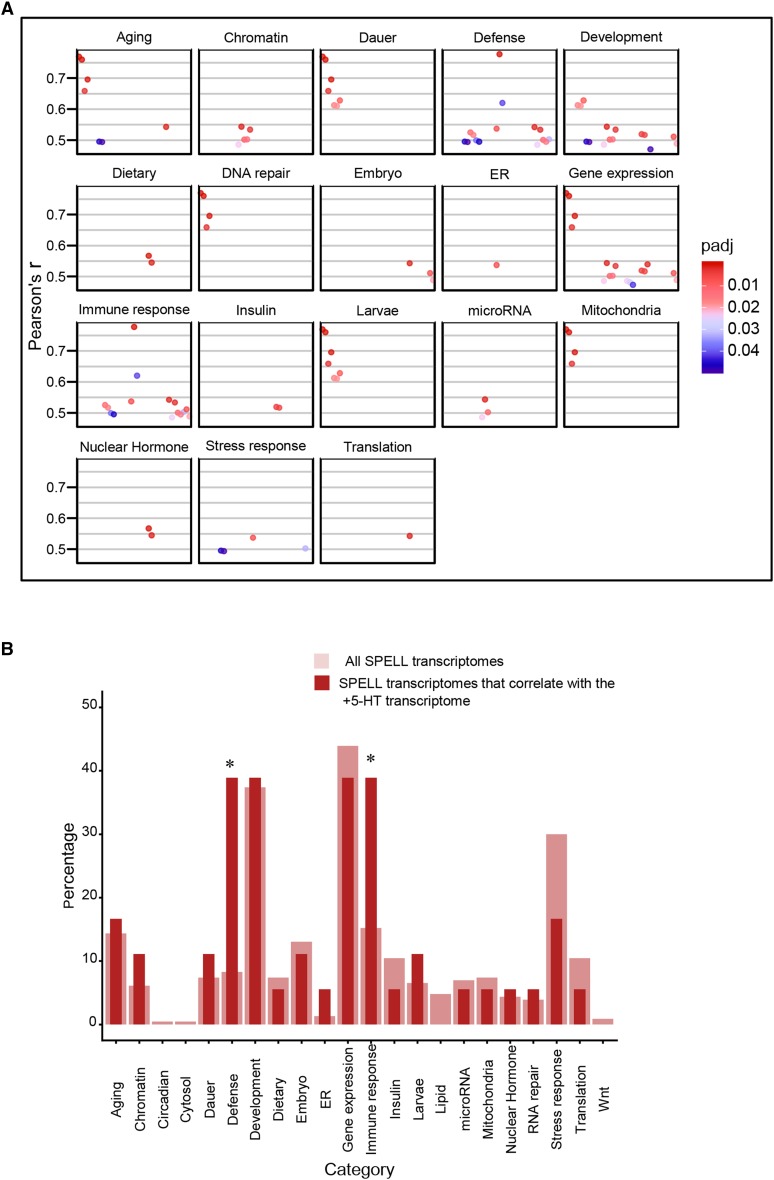
Comparison of transcriptome from 5-HT treated animals with transcriptomes in the SPELL Database. (A) Pearson’s r correlations for the datasets from the SPELL database that correlate significantly (p-adjust < 0.05) with the the 5-HT RNA-seq data. The significantly correlated SPELL datasets are grouped by category. x-axis represents individual experiments in each category, y axis the r values. (B) Bar Chart of percentage of datasets in each category. Thick light-red bars represent the percentages of the total transcriptomes from the SPELL datasets (230) that belong to a category. Thin dark-red bars represent the percent of the 18 correlating SPELL datasets that belong to that particular category. * indicates significant enrichment (*P* < 0.05; hypergeometric enrichment test).

**Figure 5:**
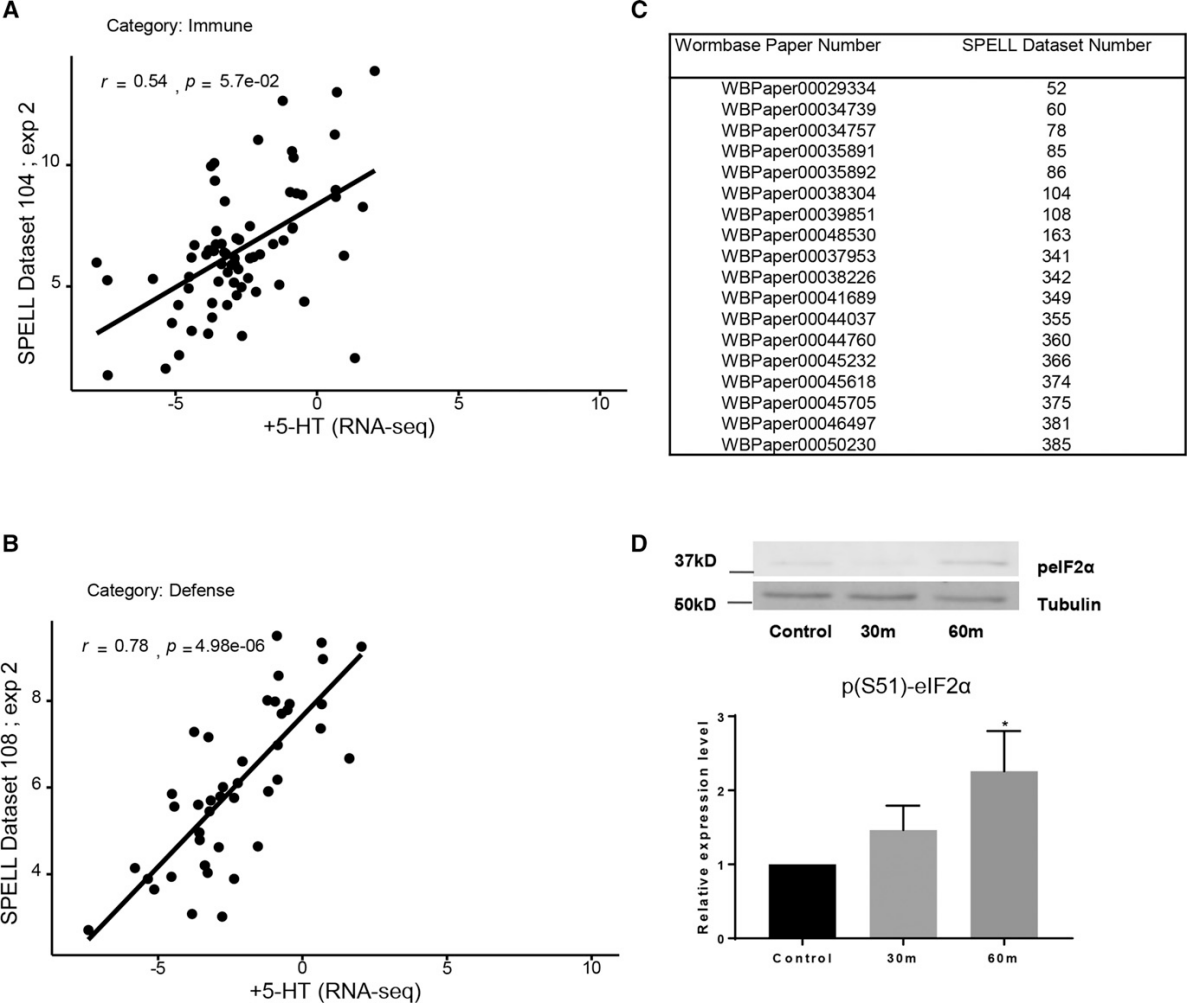
5-HT treatment induced similar changes as seen in stressed animals. (A, B) Scatterplot showing the relationship between significantly enriched genes from 5-HT-treated animals and genes from a SPELL transcription dataset from the two enriched categories. Line represents linear regression, Pearson’s r value shown. p-value is corrected for multiple tests. (A) the relationship between significantly enriched genes from 5-HT-treated animals and animals in SPELL dataset belonging to the ‘Immune’ category. (B) the relationship between significantly enriched genes from 5-HT-treated animals and animals in the SPELL dataset belonging to the ‘Defense’ category. (C) Table showing the list of datasets and publications that showed a significant correlation with the 5-HT RNA-seq data. The left column indicates the Wombase ID and the right column the SPELL ID. (D) Phospho- eIF2α (p (Ser51)-eIF2α) levels in animals with elevated 5-HT. Top Panel shows representative Western Blot using anti-phospho (Ser51)-eIF2-α antibody. Lower Panel show Bar chart of the relative expression p(Ser51)-eIF2α normalized to the untreated control. Tubulin was used as a loading control. Bars in D represent mean, error bars correspond to standard error of the mean. Significance was determined using a student’s two-tailed *t*-test. *P* < 0.05.

To obtain a clearer picture of the biological processes that were affected by an increase in 5-HT we (a) performed a Gene Ontology (GO) analysis to identify enrichment (p-value <0.01, Q-value <0.05) in the three categories: Biological Process, Molecular Function and Cellular Process, and (b) compared the RNA-seq data with compiled transcriptomes in the SPELL *C. elegans* transcription database. In animals with excess 5-HT, the differentially expressed genes (predominantly downregulated) were enriched in GO terms for extracellular matrix, cuticle and molting activities and in the negative regulation of degradation pathways (examples: negative regulation of proteolysis, negative regulation of endopeptidase activity, negative regulation of hydrolase activity, serine-type endopeptidase inhibitor etc.; Figure 3). This suggests, although does not confirm, that protein degradation pathways were upregulated upon acute increases in 5-HT. A correlation with datasets in the SPELL database showed that the 5-HT transcriptome was significantly correlated (adjusted *P* < 0.05; Pearson correlation corrected for multiple tests) with 18 datasets in the database. These datasets when categorized by keywords, clustered into groups (Figure 4A,B) from animals that had mounted a defense response (r = 0.48-0.78) or subjected to physiological stress (aging; r = 0.50-0.77 and dauer, r = 0.61-0.77), DNA damage (r = 0.66-0.77), environmental stressors such as pathogens (r = 0.48-0.78) and oxidative and heat stress (r = 0.49-0.54) and datasets dealing with mitochondria (r = 0.66-0.77), insulin-like signaling (r = 0.51) development (0.47-0.63) and chromatin (r = 0.48-0.54). Of these, the datasets that dealt with ‘defense’ and ‘immune’ were significantly enriched (*P* < 0.05). Pearson’s r between the 5-HT transcriptome and the correlated datasets from the SPELL database (Figure 4A,B), and scatter plots between representative experiments from the enriched datasets (Figure 5A,B) and the list of significantly correlated SPELL datasets and associated manuscripts are shown (Figure 5C).

Given the resemblance of the 5-HT RNA-seq dataset to transcriptomes of stressed animals, we examined whether increasing 5-HT levels either through exposure to 5-HT, or by activating release through optogenetic stimulation of 5-HT neurons, increased the levels of phosph-eIF2α, a marker of cellular stress (Kim *et al.* 2018; Kulalert *et al.* 2017; Taniuchi *et al.* 2016; Pakos-Zebrucka *et al.* 2016). This was indeed the case, and excess 5-HT caused a time-dependent increase in the levels of phosphorylated eIFα (Figure 5D).

These data together with previous studies that showed that in *C. elegans* the acute release of 5-HT activates aversive behavior and stress-gene expression, support the hypothesis that as in mammals, an acute increase in organismal 5-HT levels may also act as a defense signal in *C. elegans*. Since elevated 5-HT levels also promotes growth and mimics food abundance, we speculate that this discrepancy may be due to the differences in regimen of 5-HT exposure, as the majority of previous studies have exposed animals for longer durations and larger concentrations of exogenous 5-HT. Alternatively, these studies may be indicative of linkages between growth and stress in a multicellular organism that remain to be elucidated.

### The chronic lack of serotonin causes a net increase in gene expression in C. elegans

We also conducted Next-Generation RNA sequencing on Day 1 adult *tph-1* *(mg280)* *II* mutant animals that are chronically deficient in 5-HT (Figure 1A) (Sze *et al.* 2000). Numerous studies in *C. elegans* have utilized *tph-1* mutants to study behavior, physiology and metabolism, and the salient findings from these studies show that *tph-1* animals are deficient in modulation of behavior in response to stimuli, phenotypically resemble starved animals, dysregulate lipid metabolism and chronically activate DAF-16 (the *C. elegans* FOXO ortholog and stress-responsive transcription factor (Liang *et al.* 2006; Sze *et al.* 2000; Lee *et al.* 2011; Palamiuc *et al.* 2017; Srinivasan *et al.* 2008). We therefore expected that the *tph-1* transcriptome would resemble that of starved and metabolically dysregulated animals.

**Figure 6:**
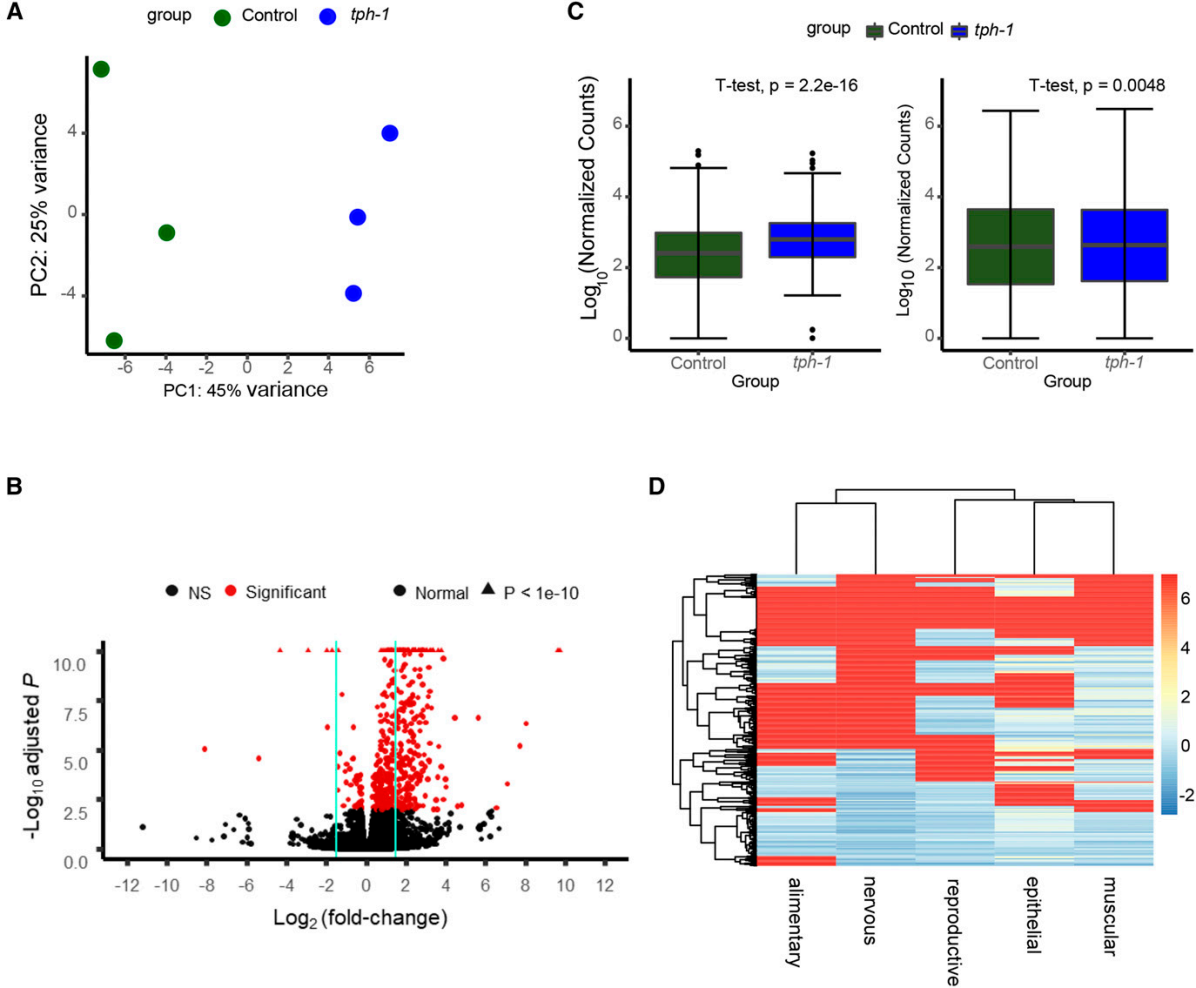
RNA-SEQ analysis reveals significant changes in expression in *tph-1* *(mg280) II* animals. (A) Principal Component Analysis (PCA) plot showing the variability between the biological replicates. (B) Volcano Plot highlighting genes with significant changes in expression (FDR-adjusted *P* < 0.01; red) or non-significant changes (black) between *tph-1* *(mg280) II* animals and control, wild-type animals. Vertical lines encompass the genes with a Log_2_(fold change) less than 1.5. In the interest of space, genes with adjusted p-value < 1e^-10^ are shown as triangles at the top of the plot. (C) Boxplots showing the differences in normalized counts per gene (log_10_ transformed) between wild type animals and *tph-1* *(mg280) II* animals. Left Panel shows the comparison between genes that were differentially expressed (FDR-adjusted *P* < 0.01). Right Panels shows the comparison of the whole transcriptome (significant and non-significant). p-value: two tailed *t*-test. Dots = outliers. (D) Cluster Heatmap showing the expression levels in different tissues of the significantly (FDR-adjusted *P* < 0.01) dysregulated genes in *tph-1* *(mg280) II* animals. Comparisons were made using data obtained from http://worm.princeton.edu. Rows and columns are clustered based on similarity in predicted expression patterns. Red-blue gradient indicates the average predicted tissue expression. red = highest predicted expression; blue = lowest predicted expression.

*tph-1* *(mg280)* *II* animals were synchronized by bleach-hatching and biological triplicates of each genotype were analyzed. The biological replicates again mostly clustering by genotype, although the wild-type replicates displayed a higher variability between samples (Figure 6A). In *tph-1* mutant animals that had grown and developed without 5-HT, 513 genes are differentially expressed compared to wild-type animals at an FDR of 0.01 (File S1; Figure 6B). Of these, in contrast to what was seen with excess 5-HT, the majority (468) of genes were upregulated, and a small fraction (45) genes were downregulated (Figure 6B,C). Most of the differentially expressed genes (246) showed changes in expression of at least 1.5-fold, and some up to 10-fold, as shown in the volcano plot (Figure 6B). A list of transcripts with the highest and lowest log-fold changes in *tph-1* are shown in tables 3 and 4. Once again, many of these genes have no clear function as annotated in Wormbase (https://www.wormbase.org/#012-34-5). Also, in contrast to what we observed with the gene set from animals with excess 5-HT, manual inspection for published pathways in which these genes were implicated did not yield a coherent picture of their function. Among the genes that displayed the largest upregulation were *T07F10.1* (*anp-1*) a poorly-characterized aminopeptidase ortholog of mammalian endoplasmic reticulum aminopeptidase (ERAP1), and ERAP2 (endoplasmic reticulum aminopeptidase 2), that by analogy to its role in other nematodes, could play roles in digestion, metabolite excretion, neuropeptide processing and/or osmotic regulation (Davey and Sommerville 1974), *fbxb-19*, *K10G4.10* and *fbxa-103* three of the 326 predicted F-box containing proteins in *C. elegans* (Kipreos and Pagano 2000) and genes predicted to encode proteins that were either transcription factors, or had DNA binding activity. Among the downregulated genes we found those that encode proteins directly involved in metabolism such as *F42A10.9*, an enzyme predicted to be involved in the breakdown of crosslinked proteins, *gfat-2* a glucosamine–fructose-6-phosphate aminotransferase predicted to catalyze the first step in the hexosamine pathway (Ghosh *et al.* 2014), *lys-10* a lysozyme involved in longevity (Samuelson *et al.* 2007), *endu-2*, an endonuclease important for stress tolerance (Ujisawa *et al.* 2018; Battisti *et al.* 2017), *acs-2* involved in fatty acid metabolism (Liang *et al.* 2010; Nomura *et al.* 2010), and *asp-8*, predicted to have endopeptidase activity. Thus, it appeared that the loss of 5-HT caused an overall increase in gene expression within the animal, as well as significantly downregulated genes that could alter metabolism. As seen with excess 5-HT tissue- expression predictions extracted using information from the website, http://worm.princeton.edu (Kaletsky *et al.* 2018) showed that the genes differentially expressed in *tph-1* mutants were also not restricted to any single tissue, but predicted to be expressed throughout the animal (Figure 6D).

**Table 3:** Upregulated Genes in *tph-1 (mg280) II* compared to wild-type

Gene	Function	Log_2_ (fold-change)	FDR
T07F10.1	Ortholog of human LNPEP, ERAP1, and ERAP2	11.41917	1.06E-74
Y40B1A.2	Ortholog of human wac	9.725711	2.67E-12
K10D3.6	Unknown	9.612714	3.31E-12
Y49F6B.13	Unknown	8.130456	2.37E-07
fbxb-19	Encodes a protein containing an F-box, a motif predicted to mediate protein-protein interactions either with homologs of yeast Skp-1p or with other proteins	7.99755	4.78E-07
scrt-1	Predicted to have nucleic acid binding activity	7.710348	6.18E-06
Y105C5A.1271	Unknown	7.133621	0.000301
F55C9.3	Unknown	7.060014	0.000506
E03A3.10	Unknown	6.95146	0.00068
hmg-6	Predicted to have DNA binding activity	6.884278	0.000885
nhr-263	Unknown	6.741746	0.000977
Y75B8A.10	Unknown	6.738259	0.002028
hpo-2	Unknown	6.521052	0.008255
C40A11.4	Ortholog of human KCTD10, KCTD13, and TNFAIP1	5.610229	2.24E-07
C24H12.3	Unknown	5.261158	0.004004
tab-1	Encodes a homeodomain protein homologous to Drosophila bsh (brain-specific homeodomain protein) and the vertebrate barh-like homeodomain protein 1	4.91197	4.2E-07
clec-33	Ortholog of human CLEC3A (and CLEC3B)	4.787472	0.005983
K10G4.10	Predicted to encode a protein with F-box domain	4.566811	0.006717
fbxa-103	Encodes a protein containing an F-box, a motif predicted to mediate protein-protein interactions either with homologs of yeast Skp-1p or with other proteins	4.434956	2.57E-07

**Table 4:** Downregulated Genes in *tph-1 (mg280) II* compared to wild-type

Gene	Function	Log_2_ (fold-change)	FDR
srw-85	Predicted to have G protein-coupled peptide receptor activity	−5.39801	3.46E-07
C08F11.7	Unknown	−4.2973	1.68E-25
C08F11.6	Unknown	−2.89479	5.54E-15
F42A10.9	Ortholog of human GGACT, predicted to have gamma-glutamylamine-cyclo-transferase activity	−1.96005	1.04E-15
nep-14	Unknown	−1.95433	7.08E-09
gfat-2	Encodes a glucosamine–fructose-6-phosphate aminotransferase (EC:2.6.1.16); GFAT-2 is predicted to catalyze the first, and rate-limiting, step of the hexosamine pathway	−1.71752	3.47E-51
lys-10	Unknown	−1.45374	2.28E-05
Y69A2AR.12	Unknown	−1.45045	1.03E-06
acs-2	Ortholog of human ACSF2 (acyl-coa synthetase 2)	−1.40768	1.7E-14
col-135	Ortholog of human COL9A2, COL19A1 (collagen type XIX alpha 1 chain), and COL9A1	−1.33963	1.58E-07
fncm-1	Unknown	−1.22663	9.81E-11
B0205.13	Encodes a small, novel protein conserved in *C. remanei* and *C. briggsae*.	−1.16146	0.000168
F09E10.1	Unknown	−1.07535	1.94E-06
asp-8	Ortholog of human PGA4, NAPSA, and PGA5, predicted to have aspartic-type endopeptidase activity	−1.02933	7.92E-06
rncs-1	Unknown	−0.99659	7.55E-07
endu-2	Ortholog of human ENDOU (endonuclease, poly(U) specific), involved in defense response to Gram-negative bacterium and innate immune response	−0.92884	7.26E-05
fbxa-192	Encodes a protein containing an F-box, a motif predicted to mediate protein-protein interactions either with homologs of yeast Skp-1p or with other proteins	−0.84533	1.35E-06
fbxa-191	Unknown	−0.79998	1.19E-05
C09G4.4	Unknown	−0.70159	3.71E-07

### The chronic lack of serotonin changes the expression of genes involved in neuronal development and transcription

**Figure 7:**
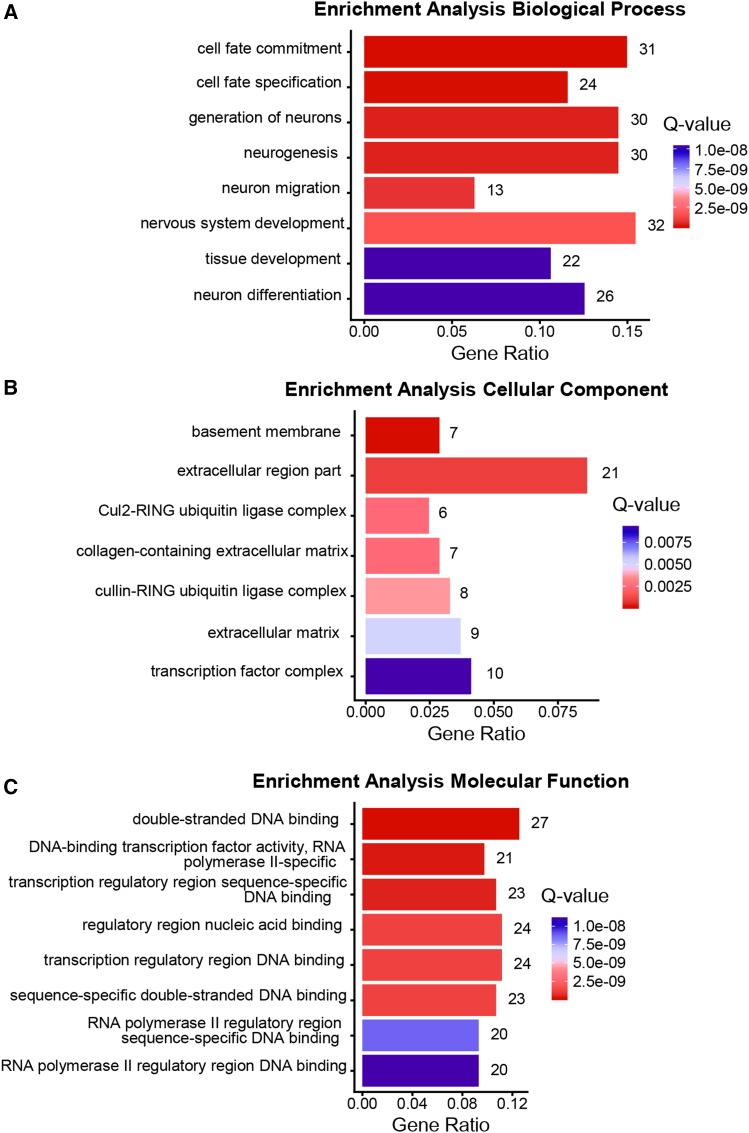
Gene Ontology of differentially expressed genes in *tph-1* *(mg280) II* animals. Top functional categories enriched in Biological Process (A), Molecular Function (B), Cellular Component (C). x axis: gene ratios. Numbers at the end of the bar indicate the number of genes present in each category; Color bar: Q-values. Red = lowest Q-values and blue = highest Q-values.

**Figure 8:**
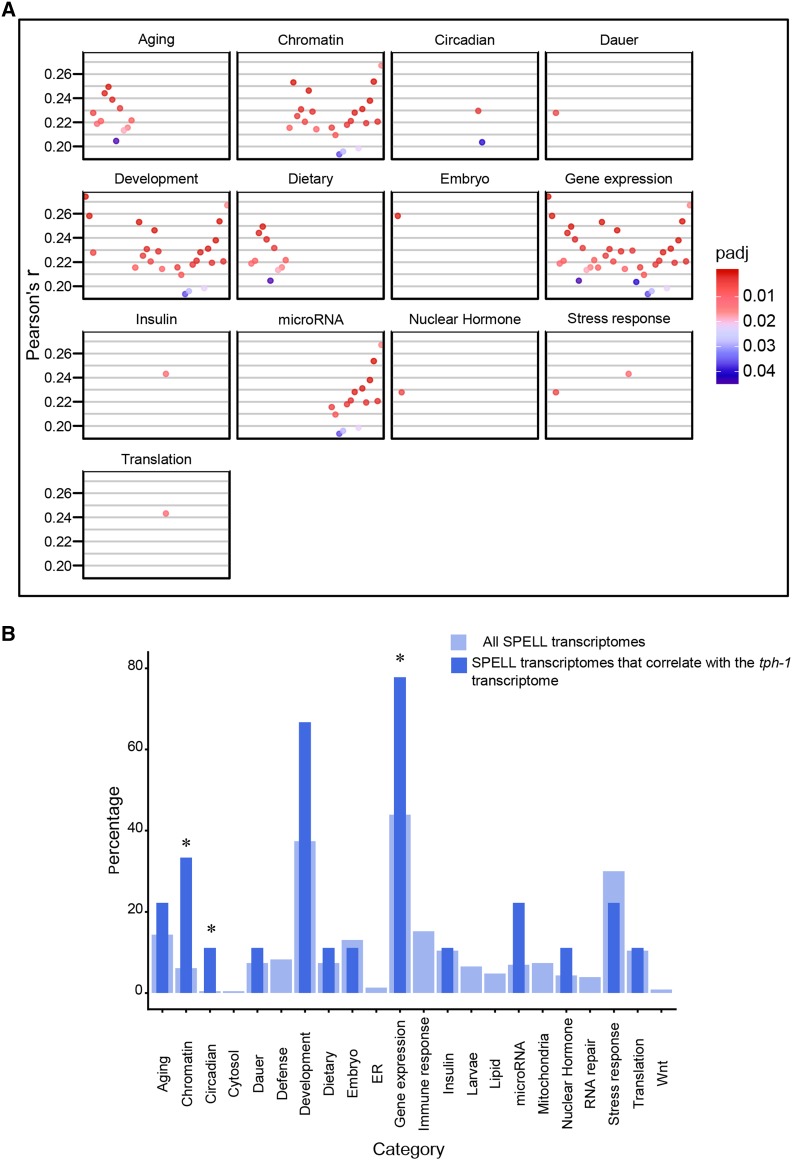
Comparison of the transcriptome of *tph-1* *(mg280) II* with SPELL Datasets. (A) Pearson’s r correlations for SPELL datasets that correlate significantly (p-adjust < 0.05) with the differential expression data from *tph-1* *(mg280) II* animals. The significantly correlated SPELL datasets are grouped by category. x-axis represents individual experiments in each category, y-axis, the r values. (B) Bar Chart of percentage of datasets in each category. Thick light-blue bars represent the percentages of the total (230) transcriptomes from the SPELL datasets. Thin dark-blue bars represent the percent of the 9 correlating SPELL datasets that belong to that particular category. * indicates significant enrichment (*P* < 0.05; hypergeometric enrichment test).

**Figure 9:**
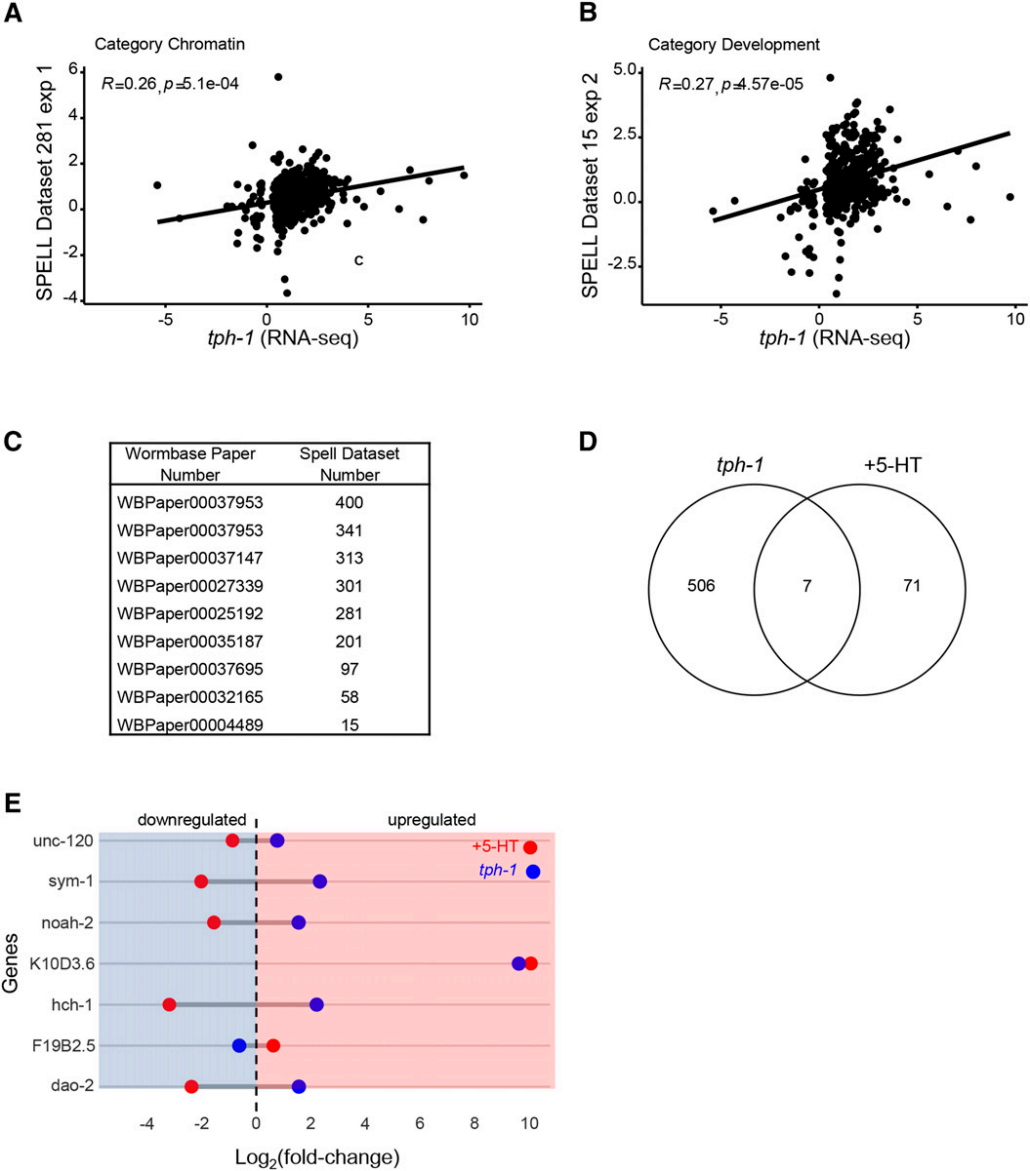
Comparison of *tph-1* with other datasets. (A, B) Scatterplot showing the relationship between significantly enriched genes in *tph-1* *(mg280) II* animals and genes from a SPELL transcription dataset from two selected categories. Line represents linear regression, Pearson’s r value shown. p-value is corrected for multiple tests. (A) the relationship between significantly enriched genes from *tph-1* *(mg280) II* animals and animals in a SPELL dataset belonging to the ‘Chromatin’ category. (B) the relationship between significantly enriched genes from *tph-1* *(mg280) II* animals and animals in the SPELL dataset belonging to the ‘Development’ category. (C) Table showing the list of datasets and publications that showed a significant correlation with the *tph-1* *(mg280) II* RNA-seq data. The left column indicates the Wombase ID and the right column the SPELL ID. (D) Venn Diagram showing overlap between the RNA-seq data from *tph-1* *(mg280) II* animals and 5-HT treated animals. (E) Dumbbell Graphs showing the Log_2_(fold-change) in expression of the genes common between 5-HT-treated animals and *tph-1* *(mg280)* *II* animals. Dotted line is at zero. Left to dotted line: downregulation (shaded in blue). Right to the dotted line: upregulation (shaded in red).

Gene Ontology (GO) analysis (p-value < 0.01, Q-value < 0.05; Figure 7A,B,C) showed an enrichment in the GO terms for Biological Processes, of genes modulating cell fate, with a predominant number modulating neuronal cell fate (neural fate determination, neuronal development, neurogenesis, and neuron differentiation; Figure 7A). This, while consistent with the role of brain 5-HT in mammalian neurodevelopment (Bonnin *et al.* 2011; Carhart-Harris and Nutt 2017), was surprising in *C. elegans*, where neuronal 5-HT impinges on all tissues. Also surprising was the dominance, among the GO terms under Molecular Function, of a large category of proteins related to DNA-binding, that included transcription factors and proteins with RNA polymerase II binding activity (Figure 7C). Among Cellular Processes genes modulating extracellular matrix components were enriched (Figure 7B). A comparison between the *tph-1* transcriptome and datasets in the SPELL transcription database yielded only 9 datasets with gene expression changes that were significantly correlated with the *tph-1* differentially expressed genes (Pearson correlation adjusted for multiple tests; *P* < 0.05; Figure 8A,B). The Pearson correlation, although significant was small (r = 0.2-0.3; Figure 9A,B,C); nevertheless, these datasets showed some congruence with the GO analysis in that datasets that were related to chromatin were significantly (*P* < 0.05) enriched, and six the 9 transcriptome datasets (66%; Pearson correlation r = 0.2-0.28) were related to development, although the enrichment for these datasets did not reach significance (*P* = 0.06). The term “gene expression”, was also enriched but is likely a non-specific keyword. Other datasets which significantly correlated with the *tph-1* transcriptome were datasets related to aging (r = 0.2-0.25), circadian rhythm (r-0.20-0.23), insulin-like signaling (r = 0.24), and nuclear hormone signaling (r = 0.22). The Pearson’s r between the genes significantly enriched in *tph-1* *(mg280) II* animals, and the datasets from the SPELL database that showed a significant correlation (Figure 8A,B), scatter plots between representative experiments and *tph-1* *(mg280) II* gene expression changes (Figure 9A,B) and the list of significantly correlated SPELL datasets are shown (Figure 9C).

Thus, *tph-1* animals did present some gene expression changes consistent with experimental studies showing a dysregulation of metabolism (Palamiuc *et al.* 2017; Rivard *et al.* 2010; Srinivasan *et al.* 2008) and insulin-like signaling (IIS)(Liang *et al.* 2006; Sze *et al.* 2000). However, in contrast to our expectation, the transcriptome changes that occur upon the chronic lack of 5-HT do not have a stress-response signature but rather, correlate with chromatin and developmental processes.

### A small but significant number of genes are differentially regulated by both the loss of 5-HT, and excess 5-HT

We examined whether both elevated 5-HT and the loss of 5-HT modulated common genes. Consistent with the two transcriptomes being obtained from animals exposed to opposing conditions (one, elevated 5-HT, and the other 5-HT deficiency) we found a very low similarity value between the samples (Jaccard Index = 0.07). Nevertheless there was a small but significant overlap (Figure 9D, *P* < 0.05) and seven genes were commonly regulated by excess 5-HT and 5-HT deficiency (Figure 9E): expression levels of all but one of these gene was downregulated in the presence of excess 5-HT and upregulated in the absence of 5-HT. These included *unc-120*, which encodes a prominent muscle transcription factor associated with motility, muscle development and aging (Mergoud Dit Lamarche *et al.* 2018), and three other genes that also modulate development: *sym-1*, which encodes a leucine-rich repeat protein with a signal sequence secreted by hypodermal cells to modulate development (Mancuso *et al.* 2012), *hch-1*, which encodes a protein related to TOLLOID and BMP-1 required for normal hatching and neuroblast migration (Hishida *et al.* 1996) and *noah-2*, which encoded a zona pellucida domain protein and was required for extracellular matrix integrity and hatching (Vuong-Brender *et al.* 2017) (Figure 9D).

### The transcriptomes of both tph-1 animals and animals with excess 5-HT overlap significantly with animals treated With the atypical antidepressant, mianserin

**Figure 10:**
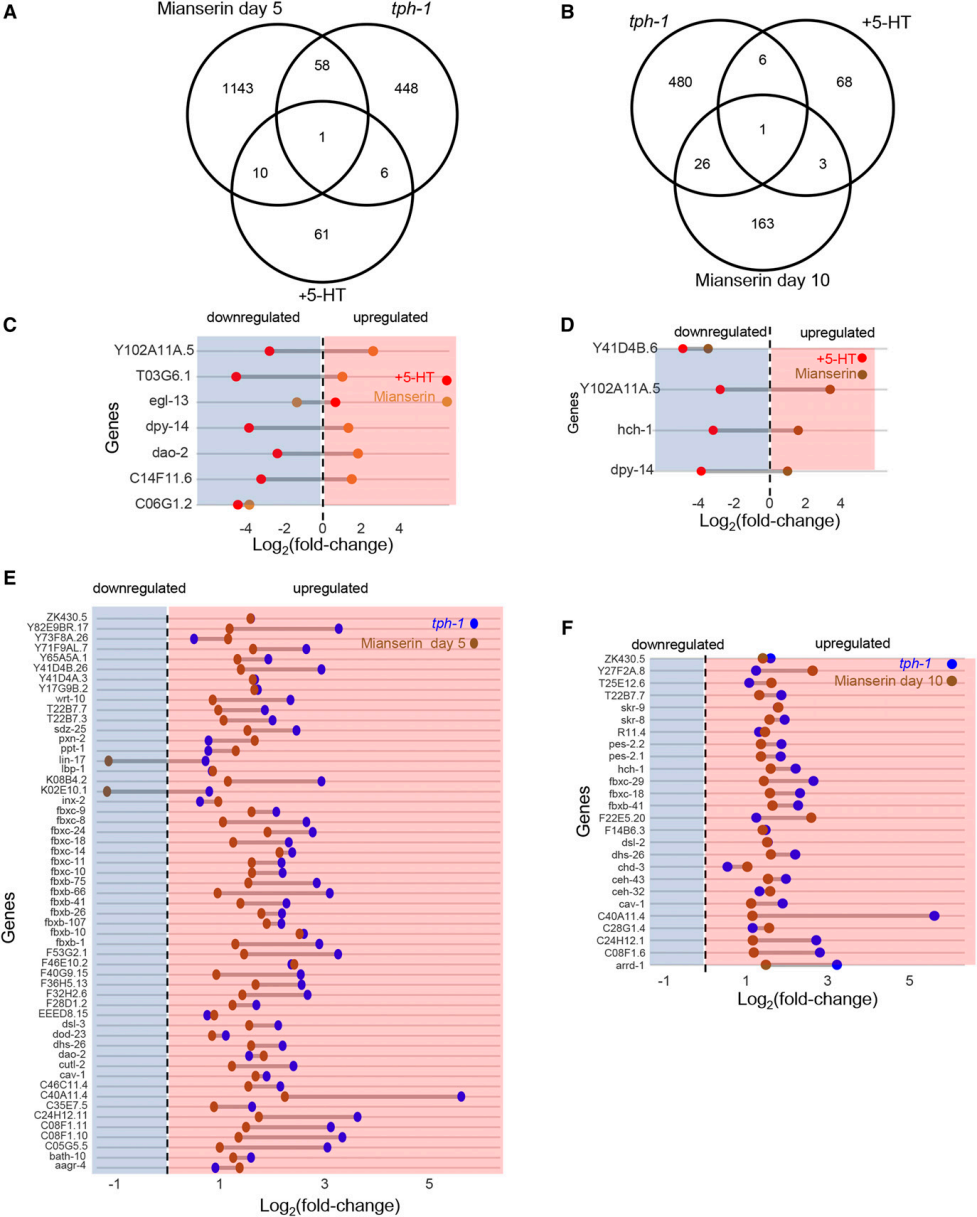
Comparison of RNA-seq data from *tph-1* *(mg280) II* animals and 5-HT treated animals with the published transcriptome of mianserin-treated animals (Rangaraju *et al.* 2015b). Venn Diagram showing overlap between *tph-1* *(mg280) II* animals, 5-HT-treated animals and (A) animals treated with mianserin for 5 days of adulthood and (B) animals treated with mianserin for 10 days of adulthood. Dumbbell Graphs showing the Log_2_(fold-change) in expression of the genes common between 5-HT-treated animals and (C) mianserin-treated animals (5 days), and (D) mianserin-treated animals (10 days). Dumbbell Graphs showing the Log_2_(fold-change) in expression of the genes common between *tph-1* *(mg280) II* animals and (E) mianserin-treated animals (5 days) and (F) mianserin-treated animals (10 days). Dotted line is at zero. Left to dotted line: downregulation (shaded in blue). Right to the dotted line: upregulation (shaded in red).

To authenticate our RNA-seq analysis we compared our RNA-seq data with the transcriptomes of animals where 5-HT levels were modulated through alternative means, namely by treatment with the 5-HT receptor antagonist, mianserin (Rangaraju *et al.* 2015a; Rangaraju *et al.* 2015b). Mianserin is used as an atypical antidepressant in humans, and increases lifespan and modulates transcriptional drift in *C. elegans* by acting through 5-HT pathways (Rangaraju *et al.* 2015a; Rangaraju *et al.* 2015b). In accordance with the role of mianserin as a 5-HT antagonist, the transcriptional changes in *tph-1* animals significantly overlapped those animals treated with mianserin for 5 and 10 days as adults (*P* = 6.95 e-5, *P* = 1.36 e-11) (Figure 10A,B). Moreover, 57 of the 59 genes and 26 the 27 genes that were common, were upregulated in both the *tph-1* mutant animals and mianserin treated animals (Figure 10E,F). The transcriptome of 5-HT treated animals and mianserin treated animals also showed a significant overlap: 11 and 4 of the 78 genes differentially regulated in the 5-HT dataset were also differentially expressed in animals treated with mianserin for 5 and 10 days respectively (*P* = 0.017, *P* = 0.01) (Figure 10A,B); however, the majority of the genes that were upregulated upon mianserin treatment were downregulated by elevated 5-HT (and vice versa; Figure 10C,D).

### The transcriptomes of tph-1 mutant animals and animals treated with exogenous 5-HT do not conform to expectations from the physiological effects of alterations in 5-HT

**Figure 11:**
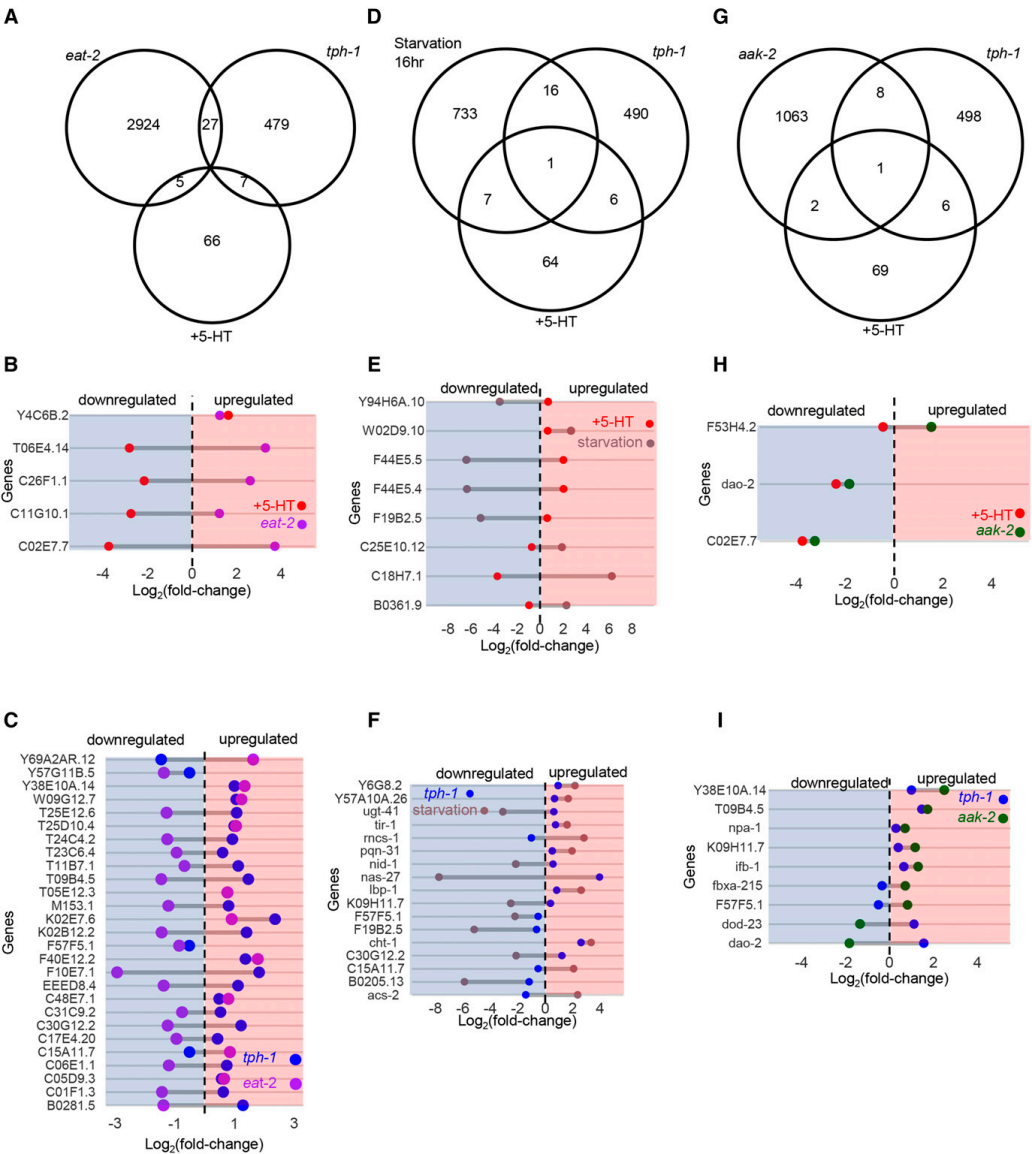
Comparison of RNA-seq data from *tph-1* *(mg280) II* animals and 5-HT treated animals with the published transcriptome of *eat-2* (*ad465*)* II* animals (Heestand *et al.* 2013), animals starved for 16 hr (Harvald *et al.* 2017), and *aak-2* (*gt33*)* X* animals (Shin *et al.* 2011) (A) Venn Diagram showing overlap between *tph-1* *(mg280) II* animals, 5-HT-treated animals and *eat-2* (*ad465*)* II* animals. Dumbbell Graphs showing the Log_2_(fold-change) in expression of the genes common between (B) 5-HT-treated animals and *eat-2* (*ad465*)* II* animals, and (C) *tph-1* *(mg280) II* animals and *eat-2* (*ad465*)* II* animals. (D) Venn Diagram showing overlap between *tph-1* *(mg280) II* animals, 5-HT-treated animals and starved animals (wild type, N2). Dumbbell Graphs showing the Log_2_(fold-change) in expression of the genes common between (E) 5-HT-treated animals and starved animals, and (F) *tph-1* *(mg280) II* animals and starved animals. (G) Venn Diagram showing overlap between *tph-1* *(mg280) II* animals, 5-HT-treated animals and published *aak-2* (*gt33*)* X* data. Dumbbell Graphs showing the Log_2_(fold-change) in expression of the genes common between (H) 5-HT-treated animals and *aak-2* (*gt33*)* X* animals, and (I) *tph-1* *(mg280) II* animals and *aak-2* (*gt33*)* X* animals. Dotted line is at zero. Left to dotted line: downregulation (shaded in blue). Right to the dotted line: upregulation (shaded in red).

Numerous studies have noted the phenotypic similarities between *tph-1* mutant animals and animals that have been deprived of food (Chao *et al.* 2004; Cunningham *et al.* 2014; Diana *et al.* 2017; Entchev *et al.* 2015; Nuttley *et al.* 2002; Sze *et al.* 2000; Srinivasan *et al.* 2008). Therefore, we directly tested the overlap between differentially expressed genes in *tph-1* animals and published transcriptome datasets from *eat-2* (*ad465*) mutants (Heestand *et al.* 2013) that have a frank decrease in food intake, and animals starved for 16 hr. (Harvald *et al.* 2017). Surprisingly, there was no significant overlap between the 513 differentially expressed genes in *tph-1* mutant animals and the over 3,000 genes differentially expressed in *eat-2* (*ad465*) mutants (Figure 11A,C *P* = 1) or the approximately 3,000 genes differentially expressed in animals starved for 16 hr (Figure 11D,F, *P* = 0.89). Moreover, of the genes common between *tph-1* mutant and the *eat-2* mutants or starved animals, nearly half were modulated in opposing directions (17 of the 27 genes common between *tph-1* and *eat-2*; Figure 11C, and 8 of 17 common between *tph-1* and animals starved for 16 hr; Figure 11F). On the other hand, the 78 genes differentially expressed upon 5-HT treatment and the approximately 3,000 genes whose expression changed in animals that had been starved for 16 hr showed a small (8 genes) but significant (*P* = 0.018) overlap (Figure 11D,E). The changes in expression levels of all but one of the 8 overlapping genes occurred in opposing directions between animals treated with excess 5-HT and starved animals (Figure 11E), consistent with the ability of exogenous 5-HT to initiate physiological responses that resemble those that occur in the presence of food.

Given the lack of correlation between the *tph-1* transcriptome and that of food-restricted animals we also examined whether the transcriptome of animals lacking *aak-2* (*gt33*) (Shin *et al.* 2011), the alpha subunit of AMP-activated kinase resembled that of animals with excess 5-HT (Figure 11G–I). This was because the loss of the neural *aak-2 *(*gt33*) in *C. elegans* has been shown to mimic the effects of elevated serotonin with respect to numerous physiological and metabolic parameters such as pharyngeal pumping rates, fat accumulation, movement, and hormonal secretions(Cunningham *et al.* 2014). Nevertheless, the 905 genes differentially expressed in *aak-2* mutant animals showed no significant overlap with the 78 genes differentially expressed in animals treated with exogenous 5-HT (Figure 11G,H, p-value = 0.86), nor did they overlap with the transcriptome of *tph-1* mutant animals (*P* = 0.99; Figure 11G,I).

These data together suggest that the phenotypic changes triggered by altering 5-HT levels may be supported by different gene expression patterns. Thus, *tph-1* mutant animals appear starved, yet did not undergo similar transcriptional changes as occur in the food-restricted animals that they resemble. This observation is consistent with previous studies that suggested that the metabolic changes in *tph-1* mutant animals may not stem from an actual decrease in food intake, but may be more related to deficiencies in the sensing or integration of signals that represent the availability of food (Gomez-Amaro *et al.* 2015). Likewise, elevated 5-HT induces physiological changes as occur in animals deficient in AMPK-dependent sensing of intracellular energy stores. Yet, the transcriptomic response to elevated levels of 5-HT, thought to promote pro-growth and differentiation pathways through inactivation of AAK-2, does not share the gene expression signature of *aak-2* deficient animals.

## Discussion

5-HT is an ancient bioamine that has profound effects on cellular and organismal processes (Azmitia 2001; Berger *et al.* 2009; Chaouloff 2002; Deakin 2013; Deakin and Graeff 1991). Popularly regarded as the ‘happiness hormone’ 5-HT signals favorable conditions under numerous contexts. For instance, it promotes the common gut microbe *Turicibacter sanguinis* to grow and thrive (Fung *et al.* 2019), promotes growth instead of virulence in *Candida* (Lass-Florl *et al.* 2003), signals the presence of food and promotes exit from dauer in *C. elegans* (Cunningham *et al.* 2014; Mylenko *et al.* 2016), and in some cases can ameliorate chronic depression in humans (Albert *et al.* 2012). Yet, in almost all organisms, elevated levels of 5-HT also signal stress (Chaouloff 2002; Chaouloff *et al.* 1999; Deakin and Graeff 1991). Serotonin modulates quorum sensing and stimulates biofilm formation in *Pseudomonas* (Knecht *et al.* 2016), a defense response of the bacterium, is synthesized and released upon exposure to abiotic stress in plants to promote abiotic stress tolerance (Erland *et al.* 2016; Kaur *et al.* 2015), promotes aversive behavior in *C. elegans* (Ooi and Prahlad 2017; Zhang *et al.* 2005), and is released as one of the early responses to real or perceived threats by mammals (Chaouloff 2002; Chaouloff *et al.* 1999; Deakin and Graeff 1991). Much of these contrary biological responses stimulated by 5-HT may have to do with the duration of exposure computed through feedback mechanisms that modulate the response of cells and animals to the presence of 5-HT at different timescales. This is most apparent in the administration of 5-HT modulators for the treatment of neuropsychiatric disorders, where an acute treatment which increase 5-HT availability cause increased anxiety, whereas chronic treatment leads to sustained antidepressant effects (Cassano *et al.* 2002; Maier 1984; Yohn *et al.* 2017). Indeed, while the effects of brain 5-HT remain mysterious in mammals, it appears that serotonergic neurons in mammals can also signal both reward and punishment (Cohen *et al.* 2015; Luo *et al.* 2016; Luo *et al.* 2015; Faulkner and Deakin 2014). Thus, acute increases in 5-HT and more prolonged increases may also trigger vastly different cellular responses.

Here we attempted to characterize the systemic changes in transcription caused by acute increases in 5-HT and the chronic deficiency of 5-HT. We find that an acute elevation of 5-HT in *C. elegans* causes transcriptional changes that, albeit small, are significant and correspond best to the transcriptional changes that occur in animals that activate a defense response to pathogens or abiotic stressors. This corresponds well with experiments that show that increasing 5-HT levels by optogenetic excitation of 5-HT producing neurons in *C. elegans* stimulates aversive behaviors and activates the stress dependent transcription factor, HSF-1. Acute increases in 5-HT also trigger an increase in pharyngeal pumping rates (ingestion of food), and a freezing response that are thought to be an indication of animals’ perception of an abundance of food. However, these latter responses may also be compatible with the activation of defense responses, as seen with the optogenetic release of 5-HT by activating dorsal raphe nucleus (DRN) 5-HT neurons in mice, which depending on the duration and circuit, induces anxiety-like behavior and suppresses spontaneous locomotion (Correia *et al.* 2017; Marcinkiewcz *et al.* 2016). Indeed, while prolonged (4 day) exposure to exogenous 5-HT is known to increase food intake and cause increases in *de novo* translation and ribosome biogenesis (Gomez-Amaro *et al.* 2015), we find that the short (30 min) duration of elevated 5-HT causes the phosphorylation of eIF2α that presumably attenuates translation. It is very possible that a short exposure of 30 min while allowing 5-HT itself to be taken up, does not allow sufficient time for translation of all the mRNA that is induced, nor allow for subsequent metabolic or physiological effects.

The main transcriptome changes seen in these RNA-seq studies on *tph-1* animals that chronically lack 5-HT are also unexpected. A GO analysis classified many of the dysregulated genes in pathways which control neuronal differentiation and transcription. In mammals, loss of normal levels of 5-HT during development changes neuronal connectivity by modulating cellular migration and cytoarchitecture, although in invertebrate and vertebrate models, animals lacking most central serotonergic neurons or neuronal serotonin synthesis enzymes can develop into adulthood without gross abnormalities in brain morphology (Daubert and Condron 2010). In *C. elegans* too, *tph-1* mutants display altered migration of specific neurons during postembryonic development, yet no gross alterations in neuroanatomy have been reported. While such gross changes may yet remain to be discovered, the changes in behavioral plasticity and learning in *tph-1* animals (Kindt *et al.* 2002), coupled with the enrichment of genes involved in neuronal development and transcription may point to the intriguing possibility that the behavioral differences in *tph-1* animals may be anchored in earlier transcriptional or developmental differences in neuronal function. In conclusion, the role of 5-HT in *C. elegans* is certainly complex. 5-HT regulates neurodevelopment and stress responsive transcriptional programs as reflected in the transcriptome data but also arousal states, memory and learning, locomotion, satiety and metabolism as seen in the numerous physiological and metabolic studies, and the relationships if any, between the different effects of this molecule, still remain to be understood.
